# Supplementary material for: Infectious bronchitis virus vaccination, but not the presence of XCR1, is correlated with large differences in chicken caecal microbiota
Source: Microb Genom. 2024 Sep 2;10(9):001289. doi: 10.1099/mgen.0.001289 (PMC11541229; doi:10.1099/mgen.0.001289)
Supplement: Uncited Supplementary Material 1. [file mgen-10-01289-s001.pdf]

Table info

Table 1: Metadata for chickens used in study.

Table 2: Description of each chicken species-level MAG (metagenome-assembled genome), including GTDB-Tk taxonomy, and CheckM2 completeness, contamination and genome quality information.

Table 3: Metabolic functional trait profiles for species-level MAGs, as output by METABOLIC.

Table 4: AMR genes identified in species-level MAGs using RGI with the Comprehensive Antibiotic Resistance Database
